# Supplementary material for: Sampling-based Continuous Optimization with Coupled Variables for RNA Design
Source: ArXiv. 2024 Dec 11:arXiv:2412.08751v1. Preprint. [Version 1] (PMC11661286)
Supplement: Supplement 1 [file NIHPP2412.08751v1-supplement-1.pdf]

Supporting Information

Sampling-based Continuous Optimization with Coupled Variables for RNA Design

Wei Yu Tang, Ning Dai, Tianshuo Zhou, David H. Mathews, and Liang Huang

S1. Derivation of Gradient

S1.A. Direct Parameterization. In the example of direct parameterization (Sec. 4.A), our objective is to compute the gradient of the objective function with respect to the parameter associated with an unpaired position i and nucleotide $\mathtt{A}$. As noted in Eq. 28, this gradient can be approximated as follows:

$$\frac{\partial \mathcal{J}(\mathbf{\Theta})}{\partial \theta^{\mathrm{u}}_{i,\mathtt{A}}} \approx \frac{1}{|\mathcal{S}|} \sum_{\boldsymbol{x} \in \mathcal{S}} \frac{\partial \log p_{\boldsymbol{y}^\star}(\boldsymbol{x}; \mathbf{\Theta})}{\partial \theta^{\mathrm{u}}_{i,\mathtt{A}}} f(\boldsymbol{x}, \boldsymbol{y}^\star)$$

Here, $\log p_{\boldsymbol{y}^\star}(\boldsymbol{x}; \mathbf{\Theta})$ can be expressed as:

$$\log p_{\boldsymbol{y}^\star}(\boldsymbol{x}; \mathbf{\Theta}) = \log \left(\prod_{i \in \mathit{unpaired}(\boldsymbol{y})} \theta^{\mathrm{u}}_{i,x_i} \cdot \prod_{(i,j) \in \mathit{pairs}(\boldsymbol{y})} \theta^{\mathrm{p}}_{i,j,x_i x_j} \cdot \prod_{(i,j) \in \mathit{mismatches}(\boldsymbol{y})} \theta^{\mathrm{m}}_{i,j,x_i x_j} \cdot \prod_{(i,j,k) \in \mathit{trimismatches}(\boldsymbol{y})} \theta^{\mathrm{tm}}_{i,j,k,x_i x_j x_k} \right)$$

$$= \sum_{i \in \mathit{unpaired}(\boldsymbol{y})} \log(\theta^{\mathrm{u}}_{i,x_i}) + \sum_{(i,j) \in \mathit{pairs}(\boldsymbol{y})} \log(\theta^{\mathrm{p}}_{i,j,x_i x_j}) + \sum_{(i,j) \in \mathit{mismatches}(\boldsymbol{y})} \log(\theta^{\mathrm{m}}_{i,j,x_i x_j}) + \sum_{(i,j,k) \in \mathit{trimismatches}(\boldsymbol{y})} \log(\theta^{\mathrm{tm}}_{i,j,k,x_i x_j x_k})$$

Since we are interested in the gradient with respect to a particular position i and nucleotide $\mathtt{A}$, the other terms are constants. This simplifies the equation to:

$$\frac{\partial \log p_{\boldsymbol{y}^\star}(\boldsymbol{x}; \mathbf{\Theta})}{\partial \theta^{\mathrm{u}}_{i,\mathtt{A}}} = \frac{\partial}{\partial \theta^{\mathrm{u}}_{i,\mathtt{A}}} (\log \theta^{\mathrm{u}}_{i,x_i}) = \frac{1}{\theta^{\mathrm{u}}_{i,x_i}} \mathbb{1}[x_i = \mathtt{A}]$$

Finally, the gradient approximation can be expressed as:

$$\frac{\partial \mathcal{J}(\mathbf{\Theta})}{\partial \theta^{\mathrm{u}}_{i,\mathtt{A}}} \approx \frac{1}{|\mathcal{S}|} \sum_{\boldsymbol{x} \in \mathcal{S}} \mathbb{1}[x_i = A] \frac{f(\boldsymbol{x}, \boldsymbol{y}^\star)}{\theta^{\mathrm{u}}_{i,\mathtt{A}}} = \frac{1}{|\mathcal{S}|} \sum_{\substack{\boldsymbol{x} \in \mathcal{S} \\ x_i = A}} \frac{f(\boldsymbol{x}, \boldsymbol{y}^\star)}{\theta^{\mathrm{u}}_{i,\mathtt{A}}}$$

We can also extend the gradient to other cases, including pairs, mismatches, and trimismatches. The gradients are listed below:

$$\frac{\partial \mathcal{J}(\mathbf{\Theta})}{\partial \theta^{\mathrm{p}}_{i,j,ab}} \approx \frac{1}{|\mathcal{S}|} \sum_{\substack{\boldsymbol{x} \in \mathcal{S} \\ x_i = a \\ x_j = b}} \frac{f(\boldsymbol{x}, \boldsymbol{y}^\star)}{\theta^{\mathrm{p}}_{i,j,ab}}, \qquad \frac{\partial \mathcal{J}(\mathbf{\Theta})}{\partial \theta^{\mathrm{m}}_{i,j,ab}} \approx \frac{1}{|\mathcal{S}|} \sum_{\substack{\boldsymbol{x} \in \mathcal{S} \\ x_i = a \\ x_j = b}} \frac{f(\boldsymbol{x}, \boldsymbol{y}^\star)}{\theta^{\mathrm{m}}_{i,j,ab}}, \qquad \frac{\partial \mathcal{J}(\mathbf{\Theta})}{\partial \theta^{\mathrm{tm}}_{i,j,k,abc}} \approx \frac{1}{|\mathcal{S}|} \sum_{\substack{\boldsymbol{x} \in \mathcal{S} \\ x_i = a \\ x_j = b \\ x_k = c}} \frac{f(\boldsymbol{x}, \boldsymbol{y}^\star)}{\theta^{\mathrm{tm}}_{i,j,k,abc}}$$

S1.B. Softmax Parameterization. In Section 4.B, we introduce the softmax parameterization as an alternative approach to solving the optimization problem. The probability of a nucleotide $a \in \mathcal{N}$ at an unpaired position i is defined in Eq. 30 as:

$$p^{\mathrm{u}}_i(a; \boldsymbol{\theta}^{\mathrm{u}}_i) \triangleq \frac{\exp(\theta^{\mathrm{u}}_{i,a})}{Z}, \quad \text{where } Z = \sum_{a' \in \mathcal{N}} \exp(\theta^{\mathrm{u}}_{i,a'})$$

To compute the gradient of the objective function under the softmax parameterization (Eq. 32), we need to derive the derivative of the softmax function. Suppose that we are interested in the derivative of $p^{\mathrm{u}}_i(a; \boldsymbol{\theta}^{\mathrm{u}}_i)$ with respect to the parameter $\theta^{\mathrm{u}}_{i,\mathtt{A}}$. For the case where $a = \mathtt{A}$, applying the quotient rule yields:

$$\frac{\partial p^{\mathrm{u}}_i(\mathtt{A}; \boldsymbol{\theta}^{\mathrm{u}}_i)}{\partial \theta^{\mathrm{u}}_{i,\mathtt{A}}} = \frac{\exp(\theta^{\mathrm{u}}_{i,\mathtt{A}})S - \exp(\theta^{\mathrm{u}}_{i,\mathtt{A}})\exp(\theta^{\mathrm{u}}_{i,\mathtt{A}})}{Z^2} = \frac{\exp(\theta^{\mathrm{u}}_{i,\mathtt{A}})}{Z} \cdot \frac{Z - \exp(\theta^{\mathrm{u}}_{i,\mathtt{A}})}{Z} = p^{\mathrm{u}}_i(\mathtt{A}; \boldsymbol{\theta}^{\mathrm{u}}_i) \cdot (1 - p^{\mathrm{u}}_i(\mathtt{A}; \boldsymbol{\theta}^{\mathrm{u}}_i))$$

Similarly, for the case where $a \neq \mathtt{A}$, the derivative is given by:

$$\frac{\partial p^{\mathrm{u}}_i(a; \boldsymbol{\theta}^{\mathrm{u}}_i)}{\partial \theta^{\mathrm{u}}_{i,\mathtt{A}}} = \frac{0 - \exp(\theta^{\mathrm{u}}_{i,\mathtt{A}})\exp(\theta^{\mathrm{u}}_{i,a})}{Z^2} = -\frac{\exp(\theta^{\mathrm{u}}_{i,\mathtt{A}})}{Z} \cdot \frac{\exp(\theta^{\mathrm{u}}_{i,a})}{Z} = -p^{\mathrm{u}}_i(\mathtt{A}; \boldsymbol{\theta}^{\mathrm{u}}_i) p^{\mathrm{u}}_i(a; \boldsymbol{\theta}^{\mathrm{u}}_i) = p^{\mathrm{u}}_i(a; \boldsymbol{\theta}^{\mathrm{u}}_i)(0 - p^{\mathrm{u}}_i(\mathtt{A}; \boldsymbol{\theta}^{\mathrm{u}}_i))$$

Combining these results, we can express the derivative of the softmax function with respect to $\theta^{\mathrm{u}}_{i,\mathtt{A}}$ as:

$$\frac{\partial p^{\mathrm{u}}_i(a; \boldsymbol{\theta}^{\mathrm{u}}_i)}{\partial \theta^{\mathrm{u}}_{i,\mathtt{A}}} = p^{\mathrm{u}}_i(a; \boldsymbol{\theta}^{\mathrm{u}}_i) \cdot (\mathbb{1}[a = \mathtt{A}] - p^{\mathrm{u}}_i(\mathtt{A}; \boldsymbol{\theta}^{\mathrm{u}}_i))$$

For other cases, such as pairs, mismatches, and trimismatches, the derivatives of the softmax function are provided below:

$$\frac{\partial p^{\mathrm{p}}_{i,j}(ab; \boldsymbol{\theta}^{\mathrm{p}}_{i,j})}{\partial \theta^{\mathrm{u}}_{i,j,a'b'}} = p^{\mathrm{p}}_{i,j}(ab; \boldsymbol{\theta}^{\mathrm{p}}_{i,j}) \cdot (\mathbb{1}[ab = a'b'] - p^{\mathrm{p}}_{i,j}(a'b'; \boldsymbol{\theta}^{\mathrm{p}}_{i,j})), \qquad \frac{\partial p^{\mathrm{m}}_{i,j}(ab; \boldsymbol{\theta}^{\mathrm{m}}_{i,j})}{\partial \theta^{\mathrm{u}}_{i,j,a'b'}} = p^{\mathrm{m}}_{i,j}(ab; \boldsymbol{\theta}^{\mathrm{m}}_{i,j}) \cdot (\mathbb{1}[ab = a'b'] - p^{\mathrm{m}}_{i,j}(a'b'; \boldsymbol{\theta}^{\mathrm{m}}_{i,j})),$$

$$\frac{\partial p^{\mathrm{tm}}_{i,j,k}(abc; \boldsymbol{\theta}^{\mathrm{tm}}_{i,j,k})}{\partial \theta^{\mathrm{u}}_{i,j,k,a'b'c'}} = p^{\mathrm{tm}}_{i,j,k}(abc; \boldsymbol{\theta}^{\mathrm{tm}}_{i,j,k}) \cdot (\mathbb{1}[abc = a'b'c'] - p^{\mathrm{tm}}_{i,j,k}(a'b'c'; \boldsymbol{\theta}^{\mathrm{tm}}_{i,j,k}))$$

Table S1. Results of different RNA Design methods on the shortest structures in Eterna100 (up to 50 and 104 nucleotides, respectively).

	Methods	Objective	$p(\boldsymbol{y}^\star \mid \boldsymbol{x})(\uparrow)$ mean	geom.†	NED$(\boldsymbol{x}, \boldsymbol{y}^\star)$ $(\downarrow)$	d(MFE$(\boldsymbol{x}), \boldsymbol{y}^\star)$ $(\downarrow)$	$\Delta\Delta G^\circ(\boldsymbol{x}, \boldsymbol{y}^\star)$ $(\downarrow)$	# of MFE $(\uparrow)$	# of uMFE $(\uparrow)$
Length ≤ 50	Matthies et al. (16)	$-\log \left[\frac{\mathbb{E}_{\boldsymbol{x}}[e^{-\Delta G^\circ(x,y)/RT}]}{\mathbb{E}_{\boldsymbol{x}}[Q(x)]}\right]$	0.545	0.088	0.120	3.72	1.12	11	11
	SAMFEO	$1 - p(\boldsymbol{y}^\star \mid \boldsymbol{x})$	0.712	0.314	**0.046**	**0.56**	**0.49**	**16**	**16**
	This Work (*projection*)	$\mathbb{E}_{\boldsymbol{x}}[-\log p(\boldsymbol{y}^\star \mid \boldsymbol{x})]$	0.715	**0.317**	0.049	0.78	0.50	15	14
	This Work (*softmax*)	$\mathbb{E}_{\boldsymbol{x}}[-\log p(\boldsymbol{y}^\star \mid \boldsymbol{x})]$	**0.716**	**0.317**	0.048	0.83	0.52	15	15
Length ≤ 104	Matthies et al. (16)	$-\log \left[\frac{\mathbb{E}_{\boldsymbol{x}}[e^{-\Delta G^\circ(x,y)/RT}]}{\mathbb{E}_{\boldsymbol{x}}[Q(x)]}\right]$	0.447	0.135	*0.110*	*6.08*	*1.38*	*27*	*27*
	SAMFEO	$1 - p(\boldsymbol{y}^\star \mid \boldsymbol{x})$	0.675	0.699	**0.038**	**1.08**	0.37	**42**	**40**
	This Work (*projection*)	$\mathbb{E}_{\boldsymbol{x}}[-\log p(\boldsymbol{y}^\star \mid \boldsymbol{x})]$	**0.681**	**0.722**	0.040	1.35	**0.33**	**42**	39
	This Work (*softmax*)	$\mathbb{E}_{\boldsymbol{x}}[-\log p(\boldsymbol{y}^\star \mid \boldsymbol{x})]$	0.680	0.717	0.041	1.43	0.34	41	**40**

(a) $p(\boldsymbol{y}^\star \mid \boldsymbol{x})(\uparrow)$ of puzzles up to 104 nucleotides.

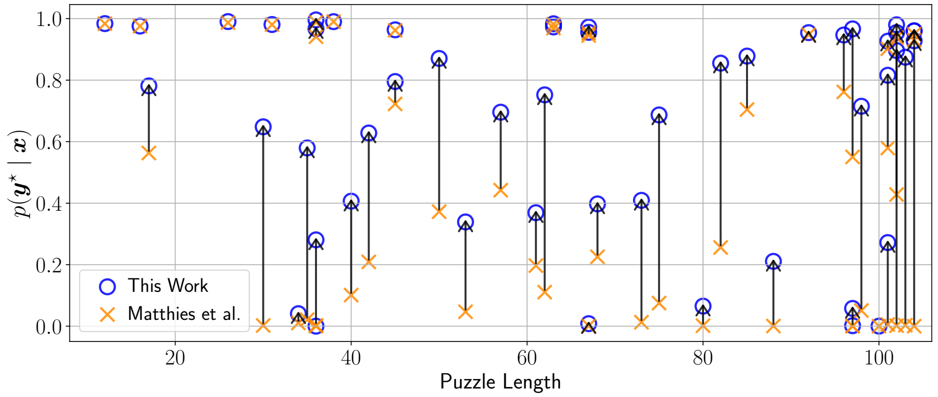

Fig. S1. $p(\boldsymbol{y}^\star \mid \boldsymbol{x})$ of solutions designed by this work vs. Matthies et al. (16) on the 51 shortest structures in Eterna100 (up to 104 nucleotides).

Table S2. Comparison of solutions designed by this work vs. SAMFEO on some long and hard-to-design Eterna100 puzzles.

Puzzle	Method	$p(\boldsymbol{y}^\star \mid \boldsymbol{x})$ $(\uparrow)$	NED$(\boldsymbol{x}, \boldsymbol{y}^\star)$ $(\downarrow)$	d(MFE$(\boldsymbol{x}), \boldsymbol{y}^\star)$ $(\downarrow)$	$\Delta\Delta G^\circ(\boldsymbol{x}, \boldsymbol{y}^\star)$ $(\downarrow)$	is MFE	is uMFE
#73 (370 nt)	This Work	**0.005**	**0.055**	**0**	**0.0 kcal/mol**	**Yes**	**Yes**
Figure 6	SAMFEO	3×10^{-27}	0.417	138	33.2 kcal/mol	No	No
#76 (393 nt)	This Work	**0.035**	**0.054**	**0**	**0.0 kcal/mol**	**Yes**	**Yes**
Figure S4	SAMFEO	7×10^{-8}	0.239	26	4.2 kcal/mol	No	No
#78 (284 nt)	This Work	**0.001**	**0.123**	**4**	**2.6 kcal/mol**	No	No
Figure 7	SAMFEO	9×10^{-25}	0.452	140	29.2 kcal/mol	No	No
#91 (392 nt)	This Work	**0.0001**	**0.034**	**8**	**3.4 kcal/mol**	No	No
Figure S5	SAMFEO	2×10^{-20}	0.128	52	25.2 kcal/mol	No	No
#99 (364 nt)	This Work	$\mathbf{8 \times 10^{-11}}$	**0.111**	**20**	**9.8 kcal/mol**	No	No
Figure S6	SAMFEO	3×10^{-28}	0.197	80	34.4 kcal/mol	No	No

Table S3. Ablation studies. Results of this work (*softmax*) optimizing for $p(y^\star \mid x)$ without mismatch and trimismatch. Targeted initialization only. †: geometric mean without 18 undesignable puzzles.

Methods	Distribution	$p(\boldsymbol{y}^\star \mid \boldsymbol{x})(\uparrow)$ mean	geom.†	NED$(\boldsymbol{x}, \boldsymbol{y}^\star)$ $(\downarrow)$	d(MFE$(\boldsymbol{x}), \boldsymbol{y}^\star)$ $(\downarrow)$	$\Delta\Delta G^\circ(\boldsymbol{x}, \boldsymbol{y}^\star)$ $(\downarrow)$	# of MFE $(\uparrow)$	# of uMFE $(\uparrow)$
This Work	v3: default	**0.589**	**0.502**	**0.035**	**2.96**	**0.81**	**78**	**75**
	v2: base-pair & mismatch	0.578	0.467	0.039	3.19	0.94	75	72
	v1: only base-pair	0.566	0.422	0.042	3.93	0.97	75	70

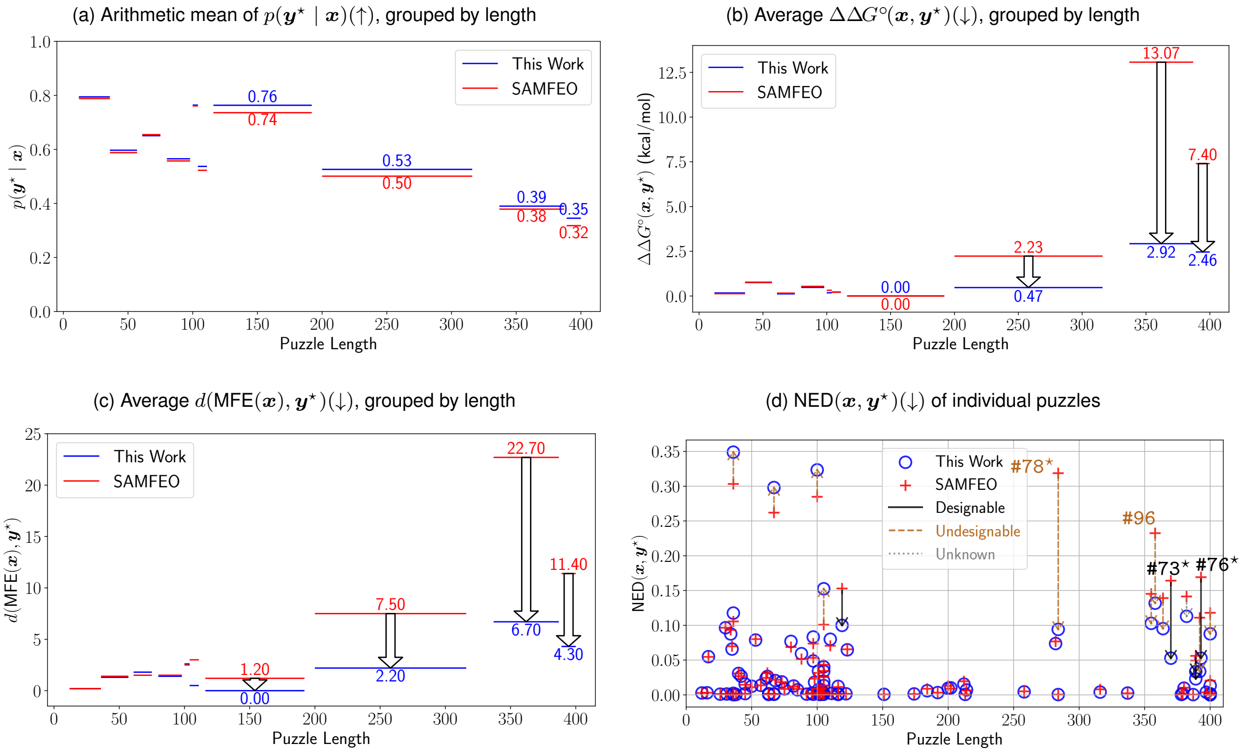

Fig. S2. (a) – (c) Average of metrics when puzzles are grouped by length, with each group consisting of 10 puzzles. (d) $\text{NED}(\boldsymbol{x}, \boldsymbol{y}^\star)$ of solutions designed by this work vs. SAMFEO. Figure 5 displays similar grouped-by-length plots and scatterplots for other metrics.

(a)

#71 (88 nt)	$p(\boldsymbol{y}^\star \mid \boldsymbol{x})$ (↑)	NED$(\boldsymbol{x}, \boldsymbol{y}^\star)$ (↓)	d(MFE$(\boldsymbol{x}), \boldsymbol{y}^\star$) (↓)	$\Delta\Delta G^\circ(\boldsymbol{x}, \boldsymbol{y}^\star)$ (↓)	is MFE	is uMFE
This Work	**0.211**	**0.059**	4	0.4 kcal/mol	No	No
NEMO	0.051	0.180	**0**	**0.0 kcal/mol**	**Yes**	**Yes**

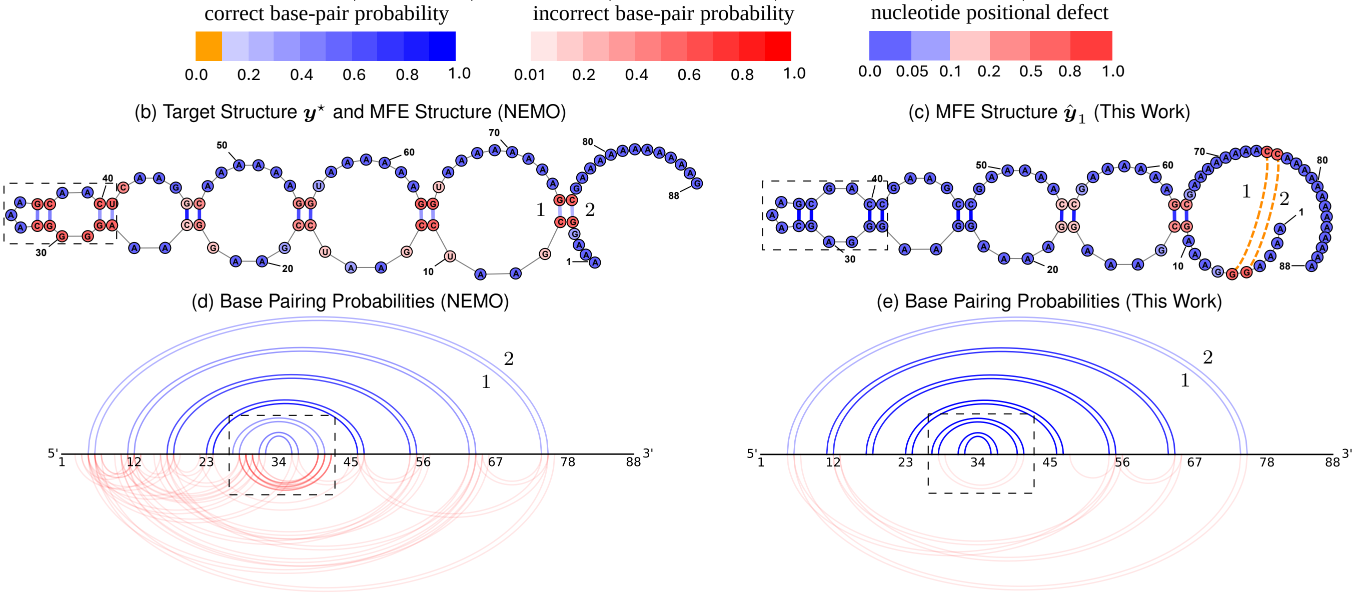

(f)

#79 (101 nt)	$p(\boldsymbol{y}^\star \mid \boldsymbol{x})$ (↑)	NED$(\boldsymbol{x}, \boldsymbol{y}^\star)$ (↓)	d(MFE$(\boldsymbol{x}), \boldsymbol{y}^\star$) (↓)	$\Delta\Delta G^\circ(\boldsymbol{x}, \boldsymbol{y}^\star)$ (↓)	is MFE	is uMFE
This Work	**0.272**	**0.041**	4	**0.0 kcal/mol**	**Yes**	No
NEMO	0.092	0.113	**0**	**0.0 kcal/mol**	**Yes**	**Yes**

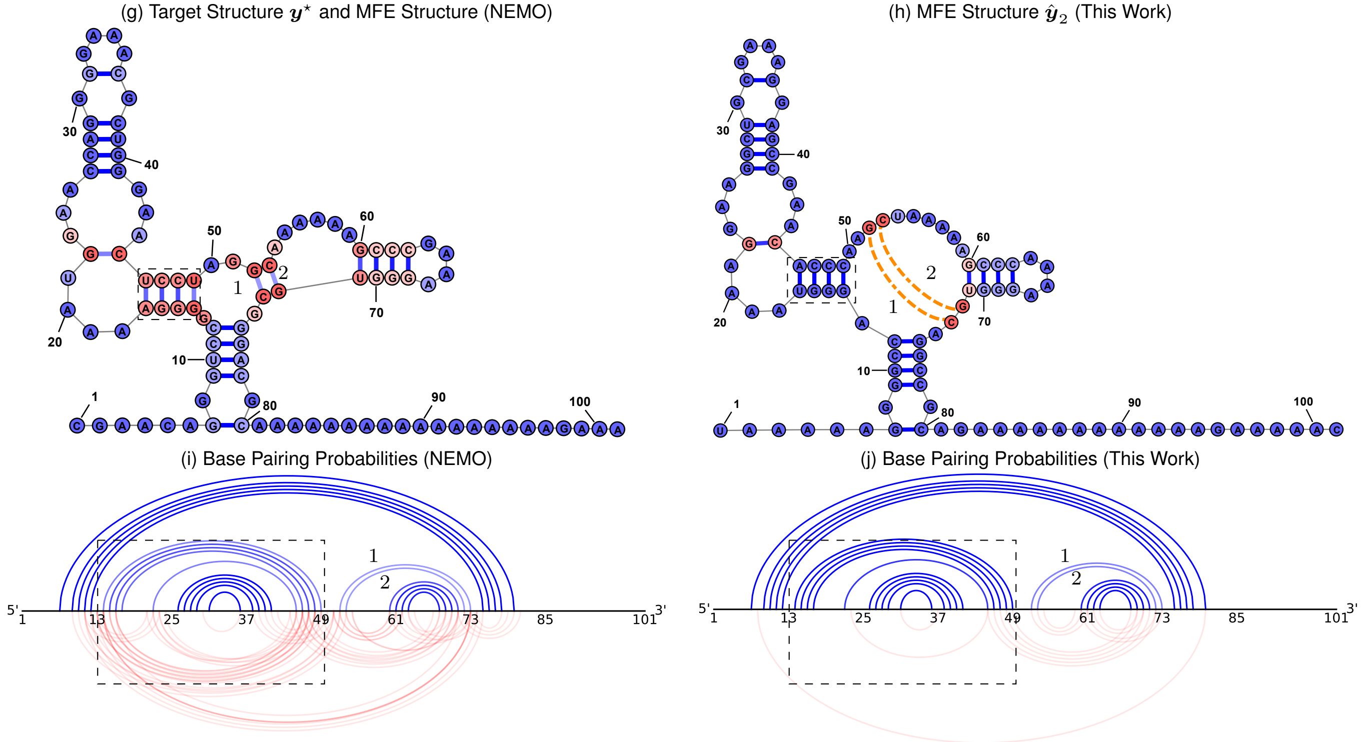

Fig. S3. Puzzles #71, #79 are solved by NEMO (but not by this work) under the UMFE criterion. MFE Structures: Base pairs are colored blue for correct pairs and red for incorrect pairs, with color intensity indicating pairing probability. Nucleotides are colored using a blue-to-red gradient to represent positional defects. Orange dashed lines in our MFE structure indicate the missing pairs from target structure. The dashed box highlights regions where NEMO's solution shows poor positional defects and many base-pairing competitions due to alternative structures. For puzzle #79, although our design is not a uMFE solution, it is still an MFE solution, meaning that $\hat{\boldsymbol{y}}_2 \in \text{MFE}_s(\boldsymbol{x})$ and $\Delta G^\circ(\boldsymbol{x}, \boldsymbol{y}^\star) = \Delta G^\circ(\boldsymbol{x}, \hat{\boldsymbol{y}}_2)$.

(a)

#76 (393 nt)	$p(\boldsymbol{y}^\star \mid \boldsymbol{x})$ (↑)	NED$(\boldsymbol{x}, \boldsymbol{y}^\star)$ (↓)	$d(\text{MFE}(\boldsymbol{x}), \boldsymbol{y}^\star)$ (↓)	$\Delta\Delta G^\circ(\boldsymbol{x}, \boldsymbol{y}^\star)$ (↓)	is MFE	is uMFE
This Work	**0.035**	**0.054**	**0**	**0.0 kcal/mol**	**Yes**	**Yes**
SAMFEO	7×10^{-8}	0.239	26	4.2 kcal/mol	No	No

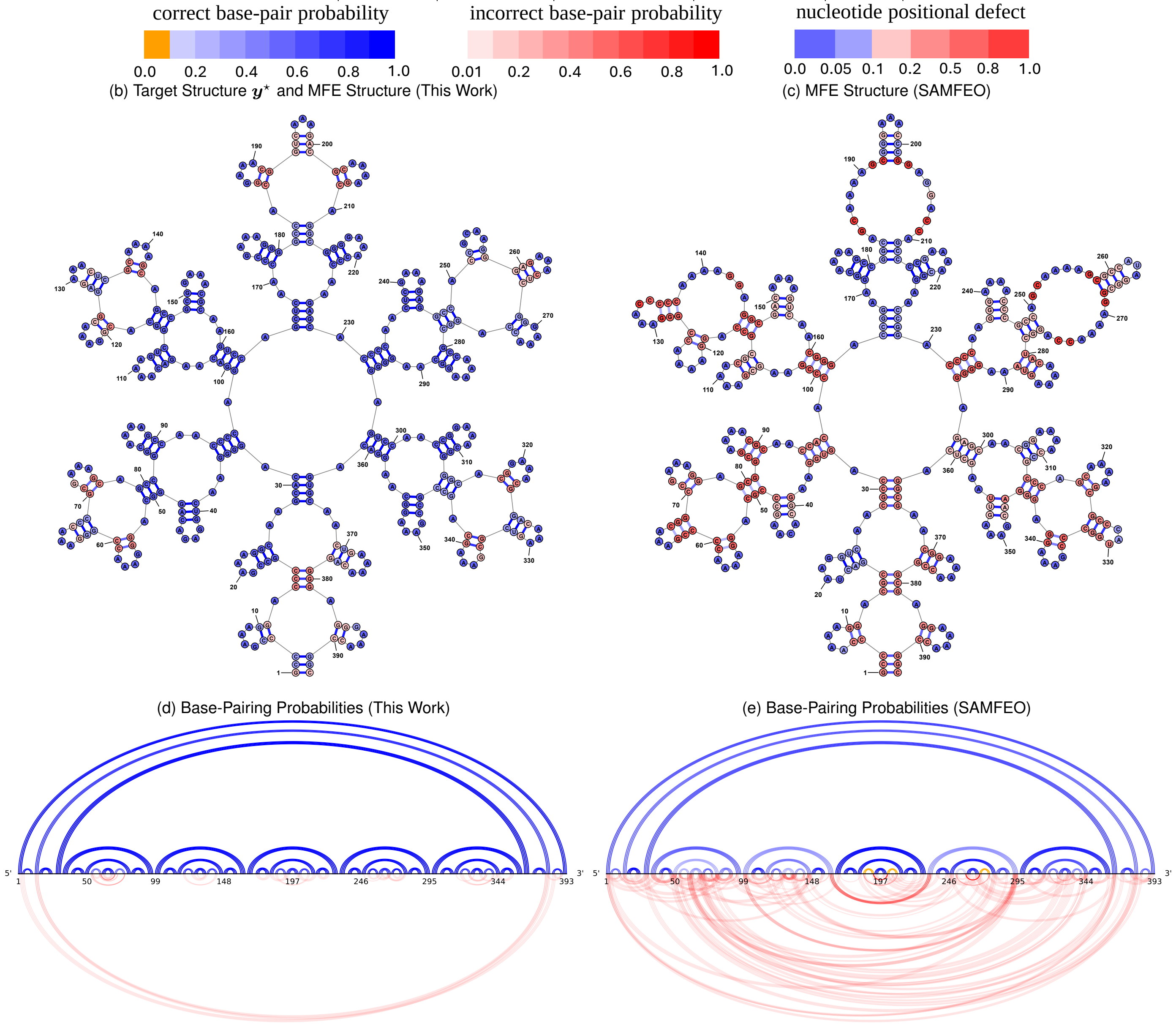

Fig. S4. Comparison of the best $p(\boldsymbol{y}^\star \mid \boldsymbol{x})$ solutions designed by this work vs. SAMFEO for Puzzle 76 ("Snowflake 3"). (b) – (c) MFE structures of the solutions from this work and SAMFEO. Base-pairs are colored as follows: blue for correct pairs, red for incorrect pairs, with the intensity indicating pairing probability. Nucleotide colors range from blue to red, indicating positional defect. (d) – (e) Base-pairing probabilities of this work and SAMFEO. Orange represents missing correct pairs (i.e. correct pairs with a pairing probability below 0.1).

(a)

#91 (392 nt)	$p(\boldsymbol{y}^\star \mid \boldsymbol{x})$ ($\uparrow$)	NED$(\boldsymbol{x}, \boldsymbol{y}^\star)$ ($\downarrow$)	d(MFE$(\boldsymbol{x}), \boldsymbol{y}^\star)$ ($\downarrow$)	$\Delta\Delta G^\circ(\boldsymbol{x}, \boldsymbol{y}^\star)$ ($\downarrow$)	is MFE	is uMFE	$p(\tilde{\boldsymbol{y}} \mid \boldsymbol{x})$ ($\uparrow$)
This Work	**0.0001**	**0.034**	**8**	**3.4 kcal/mol**	No	No	**0.037**
SAMFEO	2×10^{-20}	0.128	52	25.2 kcal/mol	No	No	8×10^{-13}

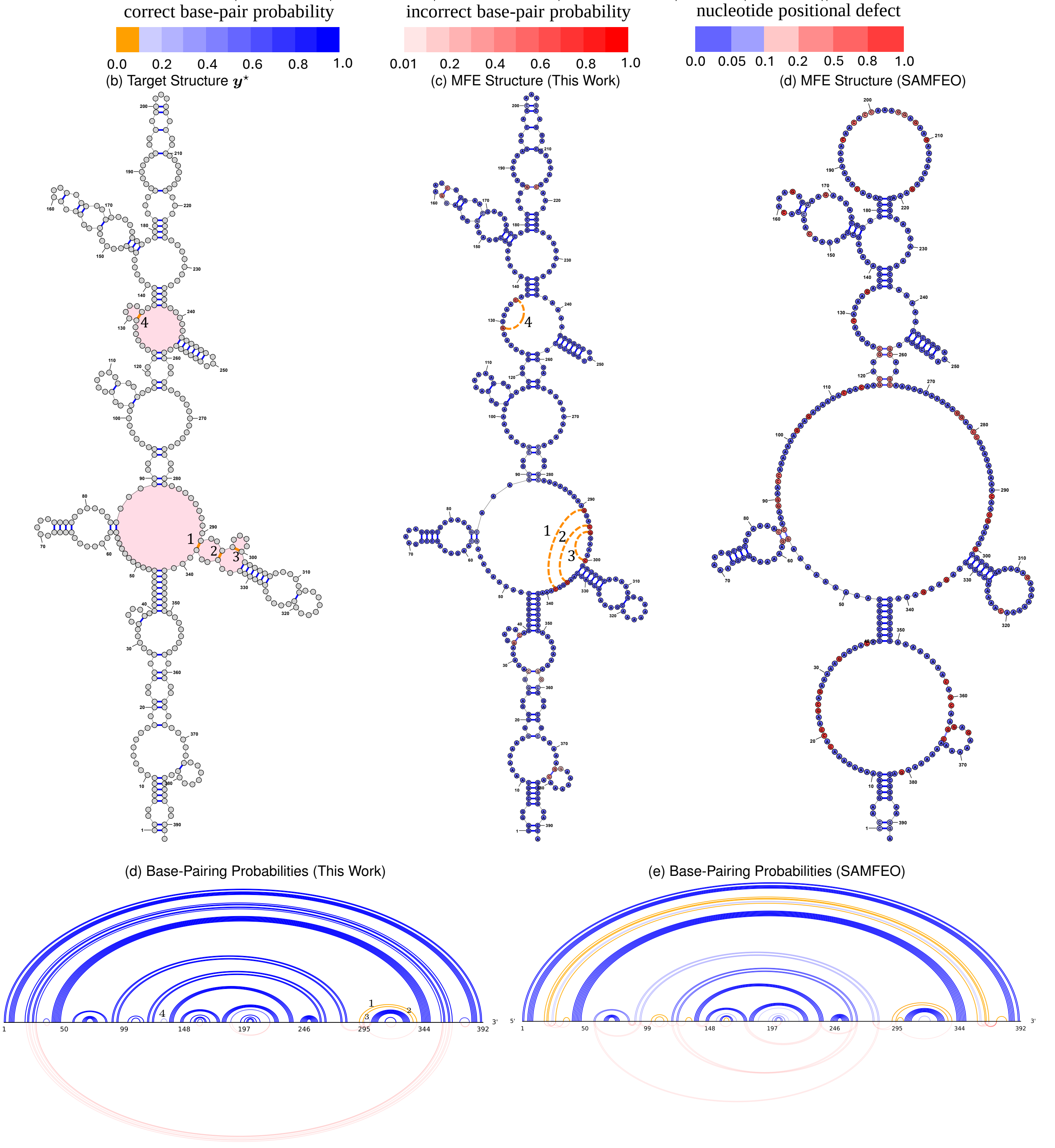

Fig. S5. Comparison of the best $p(\boldsymbol{y}^\star \mid \boldsymbol{x})$ solution designed by this work vs. SAMFEO for Puzzle 91 ("Thunderbolt"). (b) Target structure: pink-filled regions highlight loops that belong to an undesignable motif, while orange base pairs represent the missing pairs in Sampling's MFE structure. (c) – (d) MFE structures of the best $p(\boldsymbol{y}^\star \mid \boldsymbol{x})$ solutions from this work and SAMFEO. (e) – (f) Base-pairing probabilities plots. Base-pairs are colored as follows: blue for correct pairs, red for incorrect pairs, with the intensity indicating pairing probability. Orange represents missing correct pairs (i.e. correct pairs with a pairing probability below 0.1). Nucleotide colors range from blue to red, indicating positional defect. $\tilde{\boldsymbol{y}}$ refers to the target structure with the (orange) base pairs from undesignable motifs removed (i.e. pairs 1, 2, 3 and 4 are removed).

(a)

#99 (364 nt)	$p(\boldsymbol{y}^\star \mid \boldsymbol{x})$ (↑)	NED$(\boldsymbol{x}, \boldsymbol{y}^\star)$ (↓)	$d(\text{MFE}(\boldsymbol{x}), \boldsymbol{y}^\star)$ (↓)	$\Delta\Delta G^\circ(\boldsymbol{x}, \boldsymbol{y}^\star)$ (↓)	is MFE	is uMFE	$p(\tilde{\boldsymbol{y}} \mid \boldsymbol{x})$ (↑)
This Work	$\mathbf{8 \times 10^{-11}}$	**0.111**	**20**	**9.8 kcal/mol**	No	No	$\mathbf{2 \times 10^{-10}}$
SAMFEO	3×10^{-28}	0.197	80	34.4 kcal/mol	No	No	4×10^{-26}

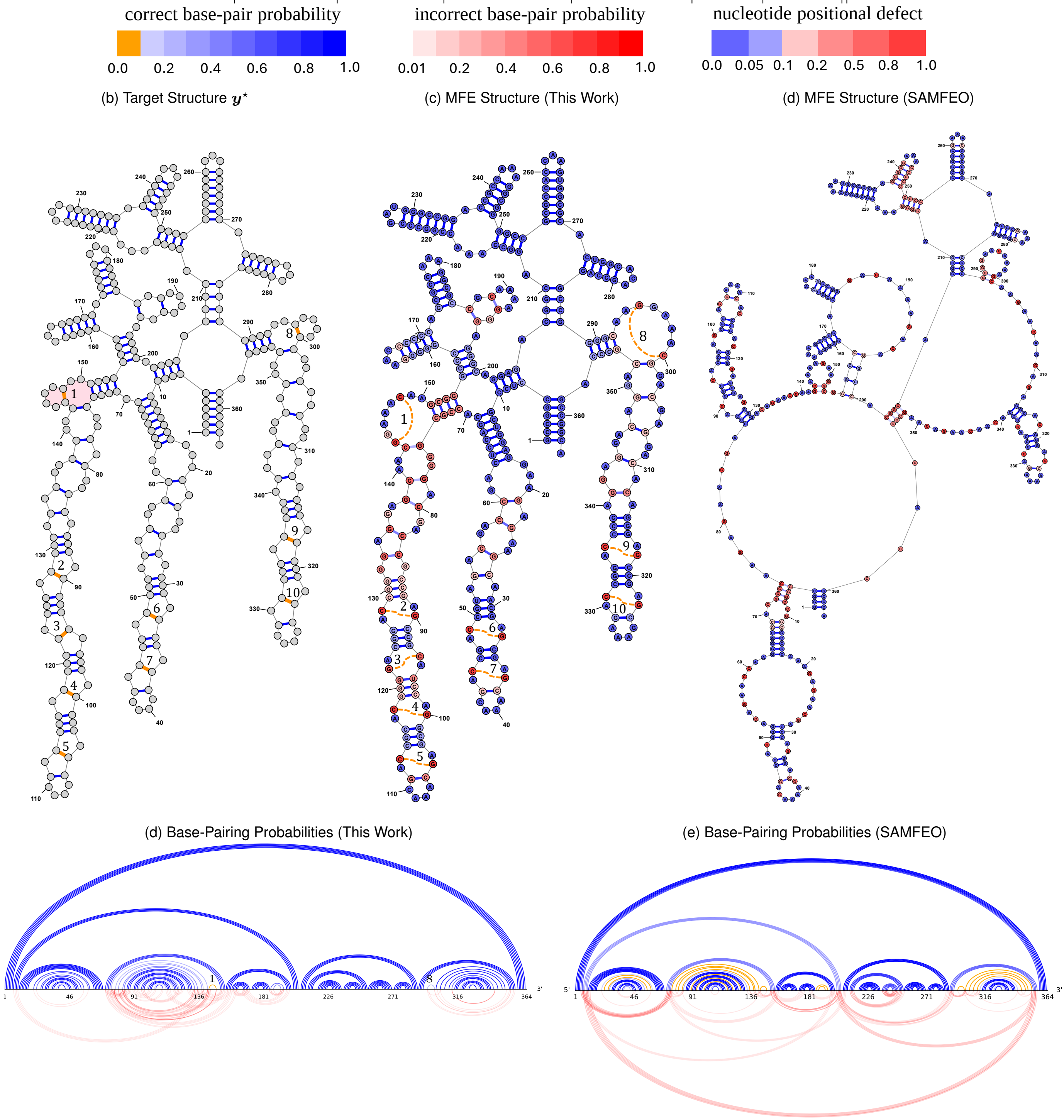

Fig. S6. Comparison of the best $p(\boldsymbol{y}^\star \mid \boldsymbol{x})$ solution designed by this work vs. SAMFEO for Puzzle 99 ("Shooting Star"). (b) Target structure: pink-filled regions highlight loops that belong to an undesignable motif, while orange base pairs represent the missing pairs in Sampling's MFE structure. (c) – (d) MFE structures of the best $p(\boldsymbol{y}^\star \mid \boldsymbol{x})$ solutions from this work and SAMFEO. (e) – (f) Base-pairing probabilities plots. Base-pairs are colored as follows: blue for correct pairs, red for incorrect pairs, with the intensity indicating pairing probability. Orange represents missing correct pairs (i.e. correct pairs with a pairing probability below 0.1). Nucleotide colors range from blue to red, indicating positional defect. $\tilde{\boldsymbol{y}}$ refers to the target structure with the (orange) base pairs from undesignable motifs removed (i.e. pair 1 is removed).

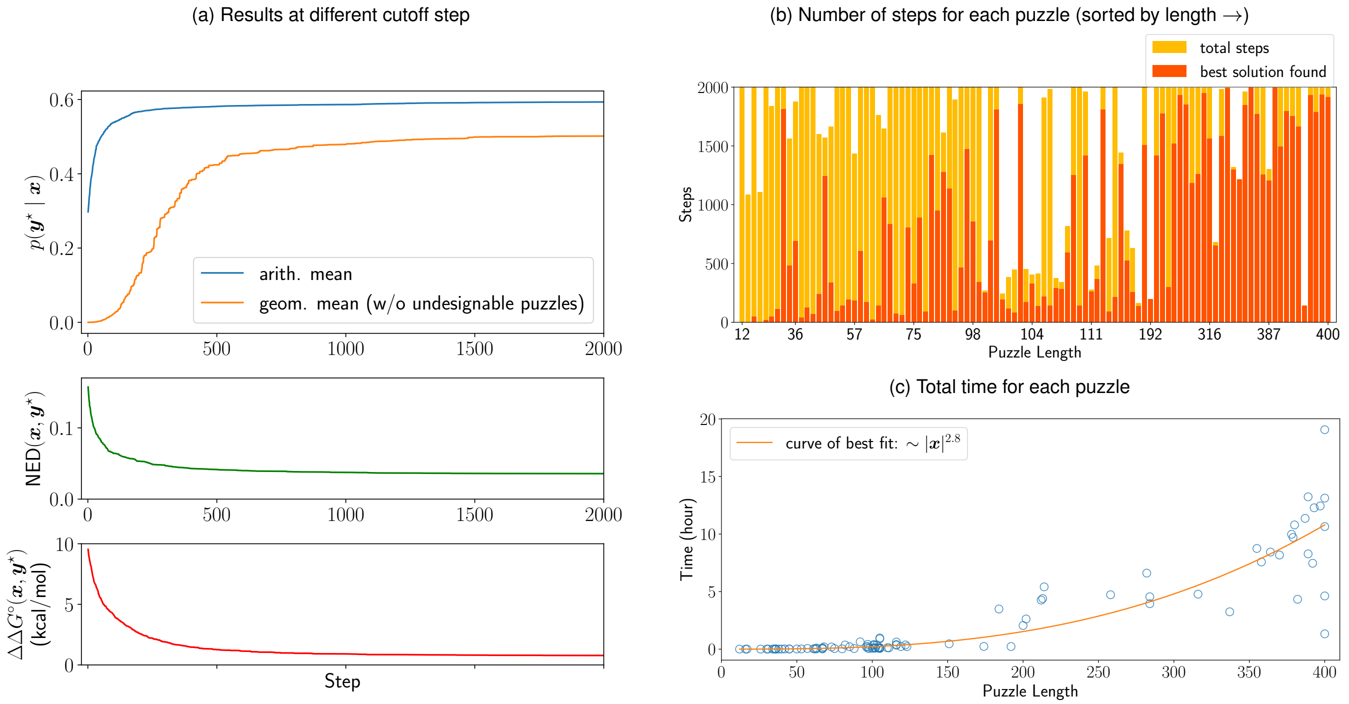

Fig. S7. (a) Metrics of the puzzles over different step cutoffs (up to 2000 steps). (b) Number of steps taken to solve each puzzle with the stopping criteria: 50 steps in which the objective function does not improved and the total number of steps is limited to 2000. (c) Total time taken to solve each puzzle, ran on a server with 28 physical cores.

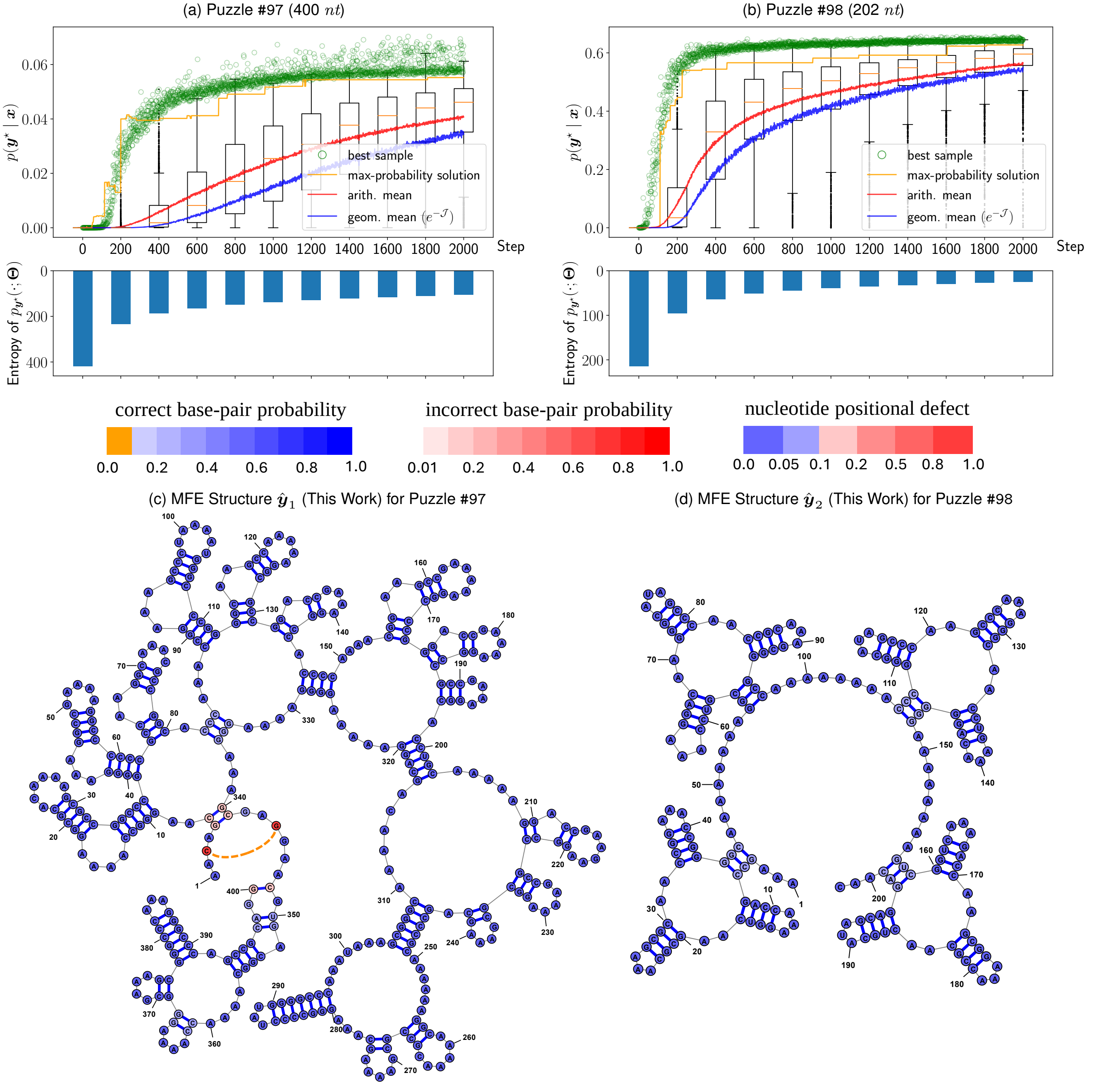

Fig. S8. (a) – (b) Learning curves of puzzles #97 and #98. Each step illustrates the $p(\boldsymbol{y}^\star \mid \boldsymbol{x})$ for both the best sample and the integral solution with the arithmetic and geometric means of $p(\boldsymbol{y}^\star \mid \boldsymbol{x})$ across all samples. The boxplots depict the distribution of $p(\boldsymbol{y}^\star \mid \boldsymbol{x})$ across all samples every 200 step, showing the interquartile range and the median of the samples. The barplots correspond to the entropy of the distribution every 200 step. (c) – (d) The MFE structures of the best $p(\boldsymbol{y}^\star \mid \boldsymbol{x})$ solution from our method. Puzzle #97 is "unknown" in the sense that it does not have a known uMFE solution and has not yet been proven to be undesignable. The MFE structure of puzzle #97, $\hat{\boldsymbol{y}}_1$, is missing a base pair from the target structure, denoted by the orange dashed line. For this puzzle, $p(\boldsymbol{y}^\star \mid \boldsymbol{x}) = 0.078$ and $p(\hat{\boldsymbol{y}}_1 \mid \boldsymbol{x}) = 0.337$.
